# Comparison of total ankle replacement and ankle arthrodesis in patients with haemophilia using gait analysis: two case reports

**DOI:** 10.1186/s13104-015-1763-y

**Published:** 2015-12-10

**Authors:** Marc Dauty, Raphael Gross, Fabien Leboeuf, Marc Trossaert

**Affiliations:** CHU Nantes, Pôle de MPR, Hôpital Saint Jacques, 85 rue Saint Jacques, 44093 Nantes Cedex 01, France; CHU Nantes, Centre Régional de Traitement de l’Hémophilie, Hôtel Dieu, 44035 Nantes Cedex 03, France

**Keywords:** Total ankle replacement, Arthrodesis, Gait analysis, Haemophilia

## Abstract

**Background:**

Severe hemophilia is an inherited, lifelong bleeding disorder characterized by spontaneous bleeding, which results in painful joint deformities. Currently two surgical treatments are available to treat haemophilia-related ankle joint destruction: ankle arthrodesis and total ankle replacement. The aim of the present study was to compare these two surgical procedures in haemophiliac subjects.

**Case presentation:**

Kinematic and dynamic parameters were quantified using a three-dimensional gait-analysis system in two similar clinical cases. In Caucasian case 1, ankle arthrodesis was chosen because of a kinematic ankle flexion defect and lack of dynamic power regeneration. The defect in energy absorption was compensated for by the contralateral side. Total ankle replacement in Caucasian case 2 allowed sparing the ipsilateral knee (maximum 0.27 preoperatively vs. 0.71 W/kg postoperatively) and hip joints powers (maximum 0.43 preoperatively vs. 1.25 W/kg postoperatively) because of the small ankle dorsiflexion motion.

**Conclusions:**

Total ankle replacement is recommended for haemophiliac patients who present with a preserved ankle range of motion.

## Background

Severe haemophilia can cause ankle arthropathy from recurrent episodes of intra-articular bleeding into the tibiotalar joint. This results in debilitating pain and irreversible destruction of joint cartilage. Once joint destruction occurs, a surgical treatment such as arthrodesis is usually performed for definitive the joint [1]. However, total ankle replacement (TAR) is a new surgical alternative that can achieve painless walking [2, 3]. We used three-dimensional (3D) gait analysis to compare two different surgical treatments, ankle arthrodesis and TAR, in two similar clinical cases. Both had presented with hemophilia-related ankle arthropathy and underwent measurement of pre and postoperative kinematic and dynamic parameters [4, 5]. Piecewise linear length normalization was used to calculate the final values [6].

## Case presentations

The arthrodesis patient was a 43-year-old Caucasian male (weight, 66 kg; height, 178 cm) with severe haemophilia B treated with human coagulation factor IX (Betafact^®^, Swedish Orphan Biovitrum AB, Stockholm, Sweden). He suffered from bilateral elbow, knee, and ankle arthropathy resulting in debilitating pain with walking [visual analog scale (VAS) score of 7/10]. The right ankle arthropathy had been treated with tibiotalar arthrodesis 5 years earlier. Preoperative right ankle range of motion (ROM) was 0° of dorsiflexion and 10° of plantarflexion. Five years postoperatively, the right surgically side was fixed at 10° of plantarflexion. Left ankle ROM was 5° of dorsiflexion and 20° of plantarflexion.

The TAR patient was a 33-year-old Caucasian male (weight, 72 kg; height, 180 cm) with severe haemophilia A, treated with recombinant factor VIII (Advate^®^, Baxalta US, Inc., Bannockburn, IL, USA), who had bilateral knee and ankle arthropathy. The patient had developed debilitating pain with walking (VAS score of 8/10) and undergone right ankle TAR 6 years earlier using a three-component Hintegra^®^ TAR system (Newdeal SA, Lyon, France). Right ankle ROM was 10° of dorsiflexion and 20° of plantarflexion preoperatively and 5° of dorsiflexion and 20° of plantarflexion 6 years postoperatively. Left ankle ROM was 20° of plantarflexion.

The knee arthropathies were similar in both cases (Pettersson score ≤5/13 for each knee). Both patients volunteered, without financial benefit, in accordance with the World Medical Association Declaration of Helsinki, to undergo a 3D gait analysis. This exam was conducted using six optoelectronic cameras (Vicon^®^ MX F40, Oxford Metrics, Oxford, UK) with a sampling rate of 100 Hz and three force plates (AMTI; Advanced Mechanical Technology, Inc., Watertown, MA, USA) with sampling rates of 1000 Hz. The marker set and mechanical conventions described by Davis et al. were used to estimate kinematics and dynamics [7]. The walking speeds were slower than standard values, with decreased step length and cadence (Table 1). The spatiotemporal parameters were symmetrical.

**Table 1:** Average spatiotemporal, ankle kinematic and dynamic parameters during six steps for arthrodesis, total ankle replacement (TAR) and comparison with Healthy subject (intra-trial confidential coefficient comprised between 0.93 and 0.98 for the left joints side and between 0.96 and 0.99 for the right joints side)

	Mr R		Mr M		Standard
	Left side	Right side (arthrodesis)	Left side	Right side (TAR)	Healthy subject
Walking speed (m/s)	1.02	1.03	0.92	0.94	1.37 ± 0.13
Cadence (step/mn)	94.4	95.2	89.8	91	109 ± 6
Step phase duration (s)	1.27	1.26	1.31	1.29	1.01 ± 0.06
Step length (m)	0.65	0.64	0.58	0.58	0.69 ± 0.05
Stance phase duration (%)	64.5	63.4	65.1	64	58.6 ± 1.8
Ankle range of motion (°)	23.1	17.1	20.8	11.9	30 ± 2
Ankle maximal and minimal power during stance phase/weight (W/kg)	1.57	0.91	1.11	0.53	2.90 ± 0.23
	−0.74	−0.36	−0.65	−0.20	−0.68 ± 0.18
Knee maximal and minimal power during stance phase/weight (W/kg)	0.05	0.71	0.06	0.27	1.80 ± 0.19
	−0.34	−0.32	−0.06	−0.23	−0.93 ± 0.12
Hip maximal and minimal power during stance phase/weight (W/kg)	0.5	1.25	0.34	0.43	0.65 ± 0.04
	−0.43	−0.31	−0.32	−0.45	−0.51 ± 0.07

### Kinematics

**Fig. 1:**
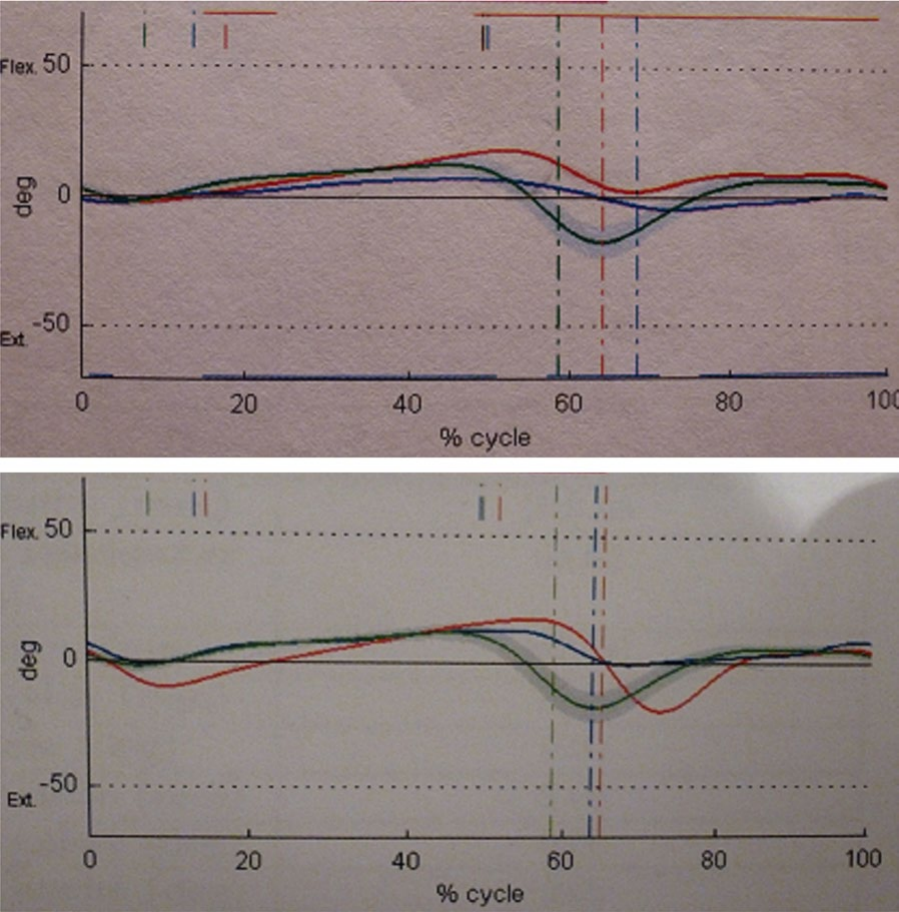
Comparison of ankle motion in the sagittal plane for the arthrodesis (of the *top*) and TAR patients (of the *bottom*) (right operated side in *blue*, left side in *red* and normal curve in *green*)

For the arthrodesis patient, 0° of dorsiflexion and 10° of plantarflexion were assumed despite tibiotalar joint fixation. ROM of the left ankle was 5° of dorsiflexion and 17° of plantarflexion. For the TAR patient, right ankle ROM of 3° of dorsiflexion and 6° of plantarflexion was assumed. Left ankle ROM was 2° of dorsiflexion and 18° of plantarflexion (Fig. 1).

### Dynamics

**Fig. 2:**
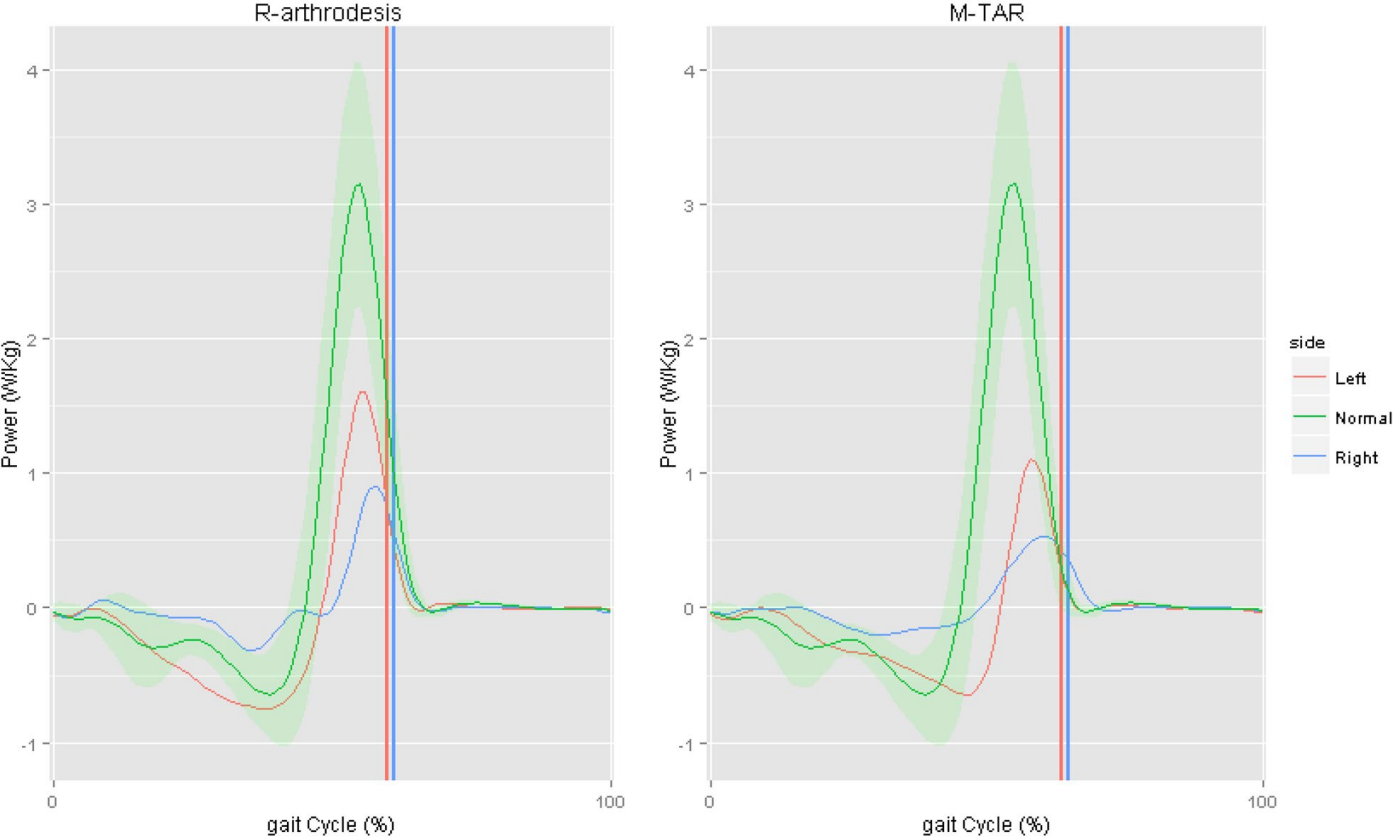
Time chart comparison of ankle power for arthrodesis and TAR. Our normal-gait database is represented in *green*, and *red* and *blue* indicate the left and right gait cycles, respectively. The *vertical lines* indicate the foot-off instants (*blue*)

In the arthrodesis patient, the right, surgically treated ankle demonstrated an absent negative phase, indicating a defect in energy absorption; a gap in the percentage of the step cycle; and a weak positive phase, demonstrating a loss of power regeneration. On the left, non-operated side, the negative phase was more prominent than normal and indicated energy absorption compensation (Fig. 2).

In the TAR patient, comparison of the operated and non-operated sides demonstrated dynamic characteristics similar to those seen in the arthrodesis patient, with decreased power in the operated ankle (0.53 vs. 0.91 W/kg) (Fig. 2).

Compensations by the knee and hip joints for decreased power in the operated ankle were greater only in ankle arthrodesis (Table 1).

## Discussion

Because of the rarity of haemophilia, no study has, to our knowledge, used gait analysis to compare surgical treatments for ankle arthropathy in hemophiliac subjects. Lobet et al. studied the consequences of ankle arthropathy in 10 patients with haemophilia. They found that, when compared with control subjects, mechanical work (i.e., strength and power) was decreased in the ankle but that walking cadence and step length were preserved because of knee and hip compensations [5]. Detrembleur et al. reported increased walking speed in 20 haemophiliac patients who underwent TAR because of the restoration of the mechanical work of the ankle, even though ankle ROM tended to decrease (22° vs. 26°) [8]. In non-haemophiliac patients with osteoarthritis, TAR has been reported to facilitate restoration of spatiotemporal and mechanical parameters to levels close to those of healthy subjects [9, 10]. However, ankle arthrodesis represents the standard for surgical treatment because of its long-term analgesic efficacy and because it has been performed for much longer than the relatively new TAR procedure. Treatments for painful haemophilia-related ankle arthropathy therefore continue to be debated.

We have reported the cases of two patients treated with arthrodesis or TAR. Reduced ankle ROM explains the change in gait pattern compared with normal controls or with results observed in the context of post-traumatic degenerative osteoarthritis [10]. Ankle arthrodesis allowed 10° of clinically measured plantarflexion, which can be understood in the context of the functional possibilities of the joints of the midfoot. This compensation could have been investigated with a specific 3D model of the foot [11] using an additional sensor at this anatomical joint to demonstrate this mobility compensation during gait analysis. On the other hand, the clinically measured ankle ROM of 20° facilitated by joint replacement was partially employed during walking. Only 9°—3° dorsiflexion and 6° of plantarflexion—were functional. In terms of dynamics, the TAR ankle generated almost half of the power generated by the arthrodesed ankle. TAR allowed sparing of ipsilateral knee and hip joint power because of the dorsiflexion facilitated, despite worse functional ROM. Long-term follow-up with clinical testing and X-ray imaging will be necessary to confirm these results, in particular for TKA [2].

## Conclusions

A difference between ankle arthrodesis and TAR in improving walking could not be demonstrated by our two clinical cases of haemophilia-related ankle arthropathy because postoperative ankle ROMs were identical. However, TAR spares ipsilateral knee and hip joint power because it enables dorsiflexion. Ankle joint replacement should therefore be recommended only when there is preserved ROM, and ankle arthrodesis when the joint is almost immobile.

## Consent

Written informed consent was obtained from both patients for publication of this case report and any accompanying images.

## Abbreviations

TAR: total ankle replacement
3D: three-dimensional
VAS: visual analog scale
ROM: range of motion
